# Effect of different lumbar–iliac fixation and sacral slope for Tile C1.3 pelvic fractures: a biomechanical study

**DOI:** 10.1186/s10195-024-00776-0

**Published:** 2024-06-26

**Authors:** Shicai Fan, Hongjie Luo, Sheqiang Chen, Haibo Xiang, Qiguang Mai, Zhenhua Zhu, Yuhui Chen, Zhiyong Hou, Wei Chen, Qingan Zhu, Yingze Zhang

**Affiliations:** 1 Department of Orthopedic Surgery, Third Hospital of Hebei Medical University, Shijiazhuang, Hebei China; 2 Department of Traumatic Surgery, Center for Orthopaedic Surgery, Third Affiliated Hospital, Southern Medical University, Guangzhou, Guangdong China; 3 Division of Spine Surgery, Department of Orthopaedics, Nanfang Hospital, Southern Medical University, Guangzhou, Guangdong China

**Keywords:** Pelvic fracture, Sacral slope, Lumbar-iliac fixation, Triangular osteosynthesis

## Abstract

**Background:**

Lumbar–iliac fixation (LIF) is a common treatment for Tile C1.3 pelvic fractures, but different techniques, including L4–L5/L5 unilateral LIF (L4–L5/L5 ULIF), bilateral LIF (BLIF), and L4–L5/L5 triangular osteosynthesis (L4–L5/L5 TOS), still lack biomechanical evaluation. The sacral slope (SS) is key to the vertical shear of the sacrum but has not been investigated for its biomechanical role in lumbar–iliac fixation. The aim of this study is to evaluate the biomechanical effects of different LIF and SS on Tile C1.3 pelvic fracture under two-legged standing load in human cadavers.

**Methods:**

Eight male fresh-frozen human lumbar–pelvic specimens were used in this study. Compressive force of 500 N was applied to the L4 vertebrae in the two-legged standing position of the pelvis. The Tile C1.3 pelvic fracture was prepared, and the posterior pelvic ring was fixed with L5 ULIF, L4–L5 ULIF, L5 TOS, L4–L5 TOS, and L4–L5 BLIF, respectively. Displacement and rotation of the anterior S1 foramen at 30° and 40° sacral slope (SS) were analyzed.

**Results:**

The displacement of L4–L5/L5 TOS in the left–right and vertical direction, total displacement, and rotation in lateral bending decreased significantly, which is more pronounced at 40° SS. The difference in stability between L4–L5 and L5 ULIF was not significant. BLIF significantly limited left–right displacement. The ULIF vertical displacement at 40° SS was significantly higher than that at 30° SS.

**Conclusions:**

This study developed an in vitro two-legged standing pelvic model and demonstrated that TOS enhanced pelvic stability in the coronal plane and cephalad–caudal direction, and BLIF enhanced stability in the left–right direction. L4–L5 ULIF did not further improve the immediate stability, whereas TOS is required to increase the vertical stability at greater SS.

## Introduction

Tile C1.3 pelvic fracture is a unilateral unstable fracture of the posterior ring, with a fracture line involving the sacrum [1, 2]. Surgical treatment is usually required to reestablish the vertical and rotational stability of the pelvic ring, otherwise serious complications, such as nonunion of fractures, loosening or rupture of internal fixation will occur [3, 4]. Because of the predominantly cancellous bone of the sacrum, weak soft tissue coverage and the high loading at the lumbosacral junction, the complication rate associated with internal fixation is high [5, 6]. There is still no clinical consensus on the treatment of Tile C1.3 [7], but lumbar–iliac fixation (LIF) is an ideal clinical treatment to restore pelvic stability because of its less invasive and proven technique. Common clinical use of LIF includes: L4–L5/L5 unilateral LIF (L4–L5/L5 ULIF), bilateral LIF (BLIF), and L4–L5/L5 triangular osteosynthesis (L4–L5/L5 TOS). However, clinicians are hesitant to choose an ideal LIF due to its high risk of surgical invasion and infection [8, 9]. Therefore, the biomechanical advantages of these LIF urgently need to be verified so as to provide references to the clinical treatments and minimize the risk.

The two-legged standing pelvis model was initially developed to evaluate posterior pelvic ring internal fixation, which can better simulate the state of human pelvis under two-legged stance [10–13]. The one-legged standing pelvis model was also established to verify anterior and posterior pelvic ring fixations in previous biomechanical studies [14–17]. However, none of these pelvic models were able to address the effects of the different morphologies of the pelvis in the standing position.

The sacral slope (SS) is an important parameter of spinal sagittal balance [18], which equals to pelvic incidence minuses pelvic tilt, and equals to lower lumbar lordosis. Larger SS leads to greater sagittal shear of the sacrum [19, 20], which may lead to internal fixation failure. However, the SS has not been thoroughly investigated for its biomechanical role in lumbar–pelvic fixation.

In this study, we developed an in vitro lumbar–pelvic loading model in two-legged standing to achieve the desired SS. The aim of this study is to evaluate the biomechanical effects of L5 ULIF, L4–L5 ULIF, L4–L5 BLIF, L5 TOS, and L4–L5 TOS for Tile C1.3 pelvic fracture under two-legged standing load at 30° and 40° SS.

## Materials and methods

### Specimens and Tile C1.3 pelvic fracture model

**Fig. 1:**
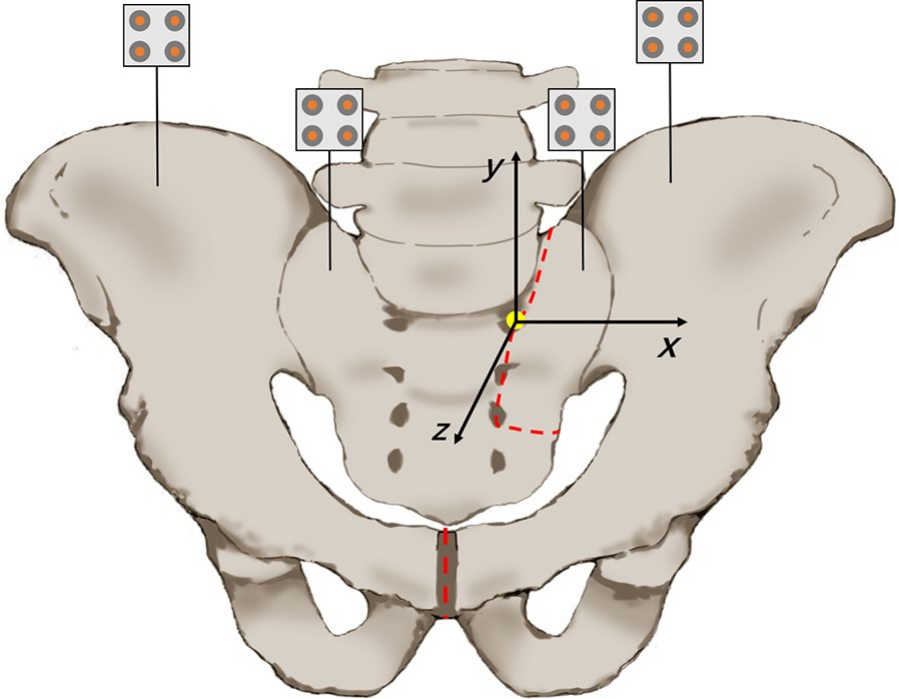
Schematic of fracture model. The red lines show the osteotomy lines. The yellow point is the virtual point and the origin of the local coordinate system. The *x* axis, *y* axis, and *z* axis represent the left–right, up–down, and front–back direction. Four scales were placed for motion tracking

This in vitro study was approved by the ethics committee. Eight fresh-frozen cadaveric male lumbar–pelvic specimens (mean age 40.5 years, range 25–65 years) were taken radiographs in anteroposterior, inlet and outlet view to rule out abnormality or injury. The specimens were stored at −20 °C and thawed at room temperature 12 h before testing. Saline was sprayed on the surface during the tests to keep the soft tissue moist. To construct Tile C1.3 pelvic fracture model, an oscillating saw blade was used to cut off the symphysis pubis and to osteotomize the posterior ring from the left S1 and S2 foramen to the left alar [21–25]. (Fig. 1).

### Fracture fixation

**Fig. 2:**
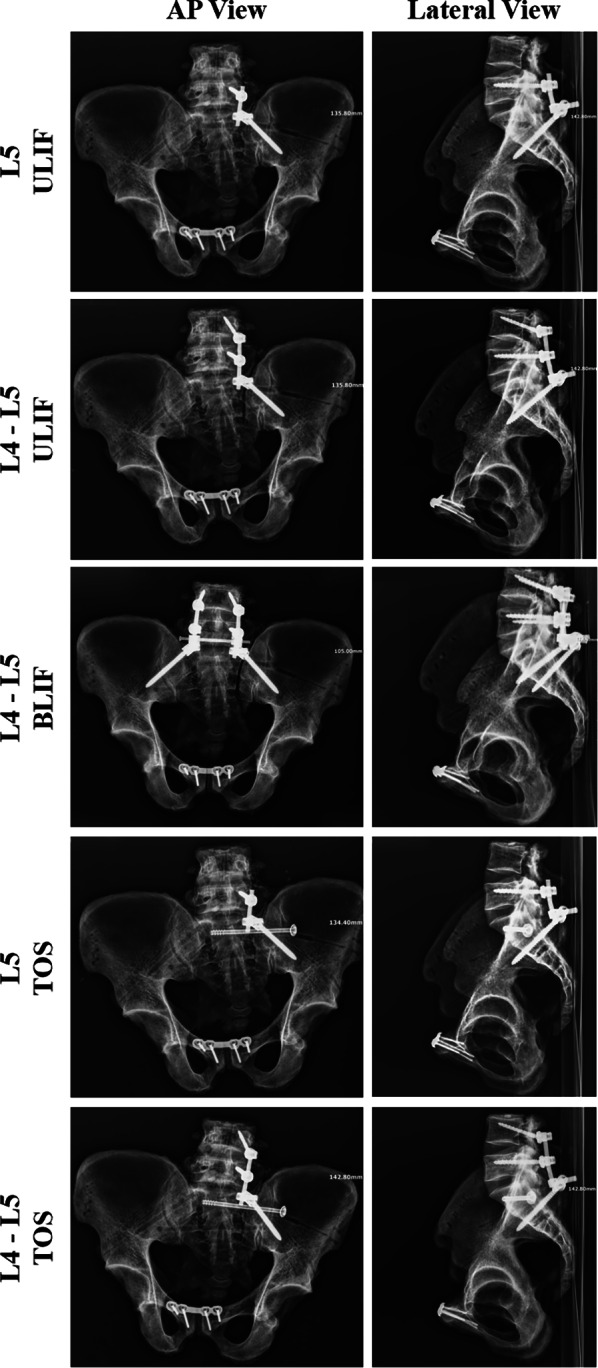
Anteroposterior and lateral pelvic X-rays of L5 ULIF, L4–L5 ULIF, L4–L5 BLIF, L5 TOS, and L4–L5 TOS. *ULIF* unilateral lumbar–iliac fixation, *BLIF* bilateral lumbar–iliac fixation, *TOS* triangular osteosynthesis

For each specimen, the anterior pelvic ring was fixed with a pubic symphyseal anatomic plate (Double Medical Technology, Inc., Xiamen, China), while the sacral fracture was fixed with five different lumbopelvic fixations (Weihai Weigao MEDICAL Devices Co., Weihai, China) in random order. The five posterior pelvic ring fixations were (1) L5 ULIF, (2) L4–L5 ULIF, (3) L4–L5 BLIF with transversal rod connection, (4) L5 TOS, and (5) L4–L5 TOS (Fig. 2). The lumbar pedicle screws and iliac screws were multiaxial screws measuring ϕ 6.5 mm × 45 mm and ϕ 7.5 mm × 80 mm, respectively. The titanium rods were 5.0 mm in diameter and the sacroiliac screws were ϕ 7.3 mm cannulated lag screws. These fixations were applied by the same skilled clinical investigator. An X-ray examination was performed to verify proper reduction and fixation.

### In vitro pelvic loading model

**Fig. 3:**
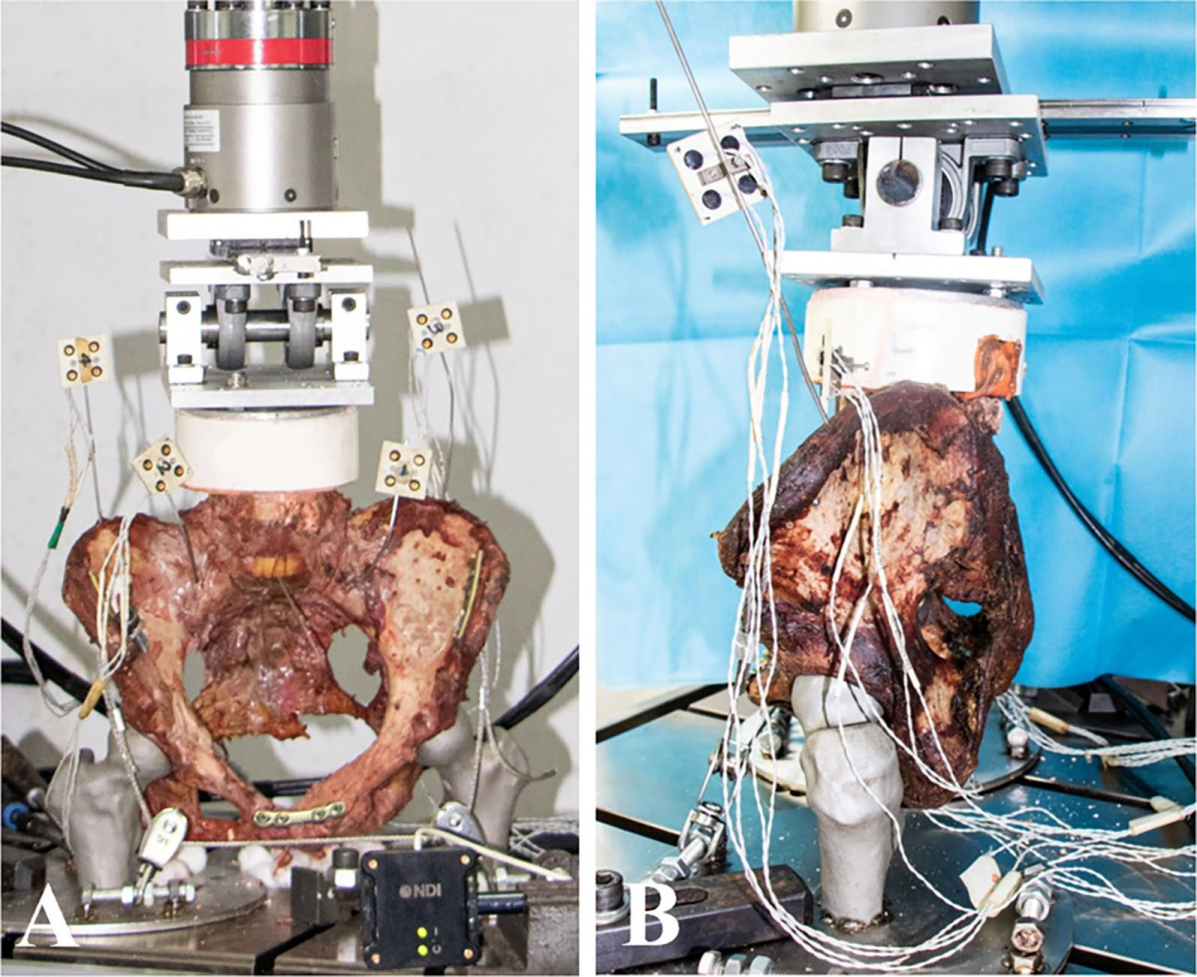
Anteroposterior (**A**) and lateral (**B**) views of experimental setup simulating pelvic motion in two-legged stance. L4 body was fixed to fixture with a pivot and a slider. Metal three-dimensional-printed femur supported the specimen. Cables and pulleys balance the pelvis. LED markers were attached to the alar and iliac crest

The superior part of the L4 body was embedded with dental stone in a PVC casting mold, keeping the L4 superior endplate horizontal. The mold was rigidly connected to a fixture, which had a transversal pivot and an anteroposterior slider, ensuring the vertical load on the L4. This structure allowed the lumbar–pelvic specimen to rotate and move during loading. (Fig. 3).

A pair of metal three-dimensional (3D)-printed proximal femurs were prepared with the femoral collodiaphyseal angle was 130° and anteversion angle was 15° and fitted into the acetabulum and secured to the platform of the material testing machine.

In addition, 2.5-mm-diameter steel cables were anchored bilaterally to the anterior superior iliac spines of the pelvic specimen and connected by two pulleys, which were fixed to the platform. The cable length changes led the entire specimen to rotate around the hip joints and adjust SS.

### Biomechanical testing

A 1.0-mm Kirschner wire was inserted parallel to the S1 endplate for SS measurement. The displacement and rotation between fracture ends at the ipsilateral S1 foramen were measured for each fixation under loading with 30° and 40° SS. A servo electromagnetic material testing machine (E10000Puls, Instron Ltd., Norwood, MA, US) applied three cycles of axial compressive force ranging from 50 to 500 N at a rate of 5 N/s to the L4 vertebrae via the loading fixture.

Kirschner wires of 1.5-mm were inserted into bilateral sacral alar and iliac crest, respectively, and each Kirschner wire was attached with four infrared LED markers. Three-dimensional motion of the markers was measured by an optotrack camera system (Certus, NDI, Waterloo, Canada). Data sampling frequency was 20 Hz in all tests.

### Data analysis

A virtual point was set as the origin of the 3D coordinate system at the left anterior foramen of S1 located in the line of the Tile C1.3 fracture (Fig. 1). The displacement and rotation were the relative movement between fracture fragments, including the displacement Tx in the left–right direction, Ty in the up–down direction, and Tz in the anterior–posterior direction, as well as flexion–extension Rx, axial-rotation Ry, and lateral-bending Rz based on the conventional spinal motion analysis, respectively. Total displacement (*T*_total_) could also be calculated.

All data analyses were performed using STATISTICA software (v.7, Dell, TX). Analysis of variance with repeated measures in a generalized linear model was used to first analyze the effects of SS (30° or 40°), additional iliosacral screw fixation (ULIF or TOS) and fixing level (L5 or L4–L5 fixation) and then the effects of fixing side (with or without traversal connector between fragments) and SS (30° or 40°). SNK was used to test differences between groups, provided that the effects of the factors were significant. The significance level was placed at *p* < 0.05.

## Results

In this study, no significant screw loosening of anterior or posterior ring fixation, or destruction of the specimens was observed. The displacement and rotational motion following each fixation were listed in Table 1.

**Table 1:** Mean and standard deviation of displacements and rotations of Tile C1.3 pelvic fracture fixations under lumbar axial compressive force

	SS	L5 ULIF	L4–L5 ULIF	L4–L5 BLIF	L5 TOS	L4–L5 TOS
*T*_total_ (mm)	40°	1.33 ± 0.50	1.34 ± 0.36	1.41 ± 0.57	0.90 ± 0.12	1.15 ± 0.33
	30°	1.21 ± 0.19	1.16 ± 0.24	1.17 ± 0.36	0.95 ± 0.12	1.05 ± 0.28
Tx (mm)	40°	0.61 ± 0.25	0.62 ± 0.19	0.51 ± 0.22	0.43 ± 0.08	0.56 ± 0.34
	30°	0.61 ± 0.20	0.60 ± 0.20	0.45 ± 0.19	0.48 ± 0.17	0.49 ± 0.22
Ty (mm)	40°	0.80 ± 0.28	0.85 ± 0.46	0.90 ± 0.52	0.43 ± 0.09	0.50 ± 0.14
	30°	0.59 ± 0.22	0.55 ± 0.30	0.53 ± 0.21	0.39 ± 0.14	0.40 ± 0.10
Tz (mm)	40°	0.84 ± 0.40	0.73 ± 0.18	0.88 ± 0.37	0.66 ± 0.08	0.83 ± 0.20
	30°	0.81 ± 0.14	0.76 ± 0.19	0.89 ± 0.37	0.68 ± 0.17	0.81 ± 0.24
Rx (°)	40°	0.59 ± 0.17	0.52 ± 0.11	0.56 ± 0.26	0.47 ± 0.13	0.58 ± 0.07
	30°	0.57 ± 0.19	0.64 ± 0.22	0.55 ± 0.18	0.53 ± 0.18	0.67 ± 0.21
Ry (°)	40°	0.97 ± 0.27	1.10 ± 0.64	0.90 ± 0.45	0.77 ± 0.27	0.70 ± 0.28
	30°	1.05 ± 0.41	1.12 ± 0.65	0.67 ± 0.17	0.88 ± 0.25	0.90 ± 0.50
Rz (°)	40°	0.84 ± 0.40	0.80 ± 0.34	0.74 ± 0.48	0.58 ± 0.34	0.57 ± 0.31
	30°	0.60 ± 0.16	0.65 ± 0.16	0.56 ± 0.16	0.56 ± 0.19	0.42 ± 0.13

Displacement is located to the ipsilateral S1 anterior sacral foramen. Rotation is located between the fractured sacra. Tx in left–right direction, Ty in vertical direction, and Tz in anterior–posterior direction. Rx in flexion–extension, Ry in axial rotation, Rz in lateral bending

*SS* sacral slope, *ULIF* unilateral lumbar–iliac fixation, *BLIF* Bilateral lumbar–iliac fixation, *TOS* triangular osteosynthesis

**Fig. 4:**
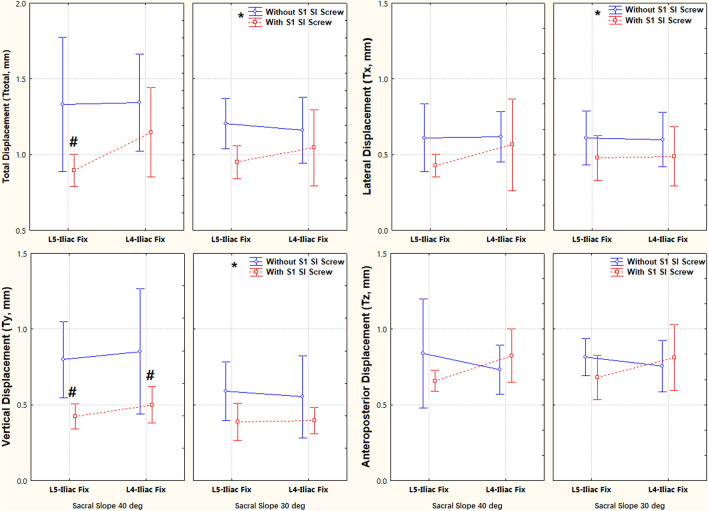
The effects of three factors, including sacral slope (30° or 40°), additional iliosacral screw fixation (ULIF or TOS) and fixing level (L5 or L4–L5 fixation) on fracture displacement. *ULIF* Unilateral lumbar–iliac fixation, *TOS* triangular osteosynthesis. Vertical bars denote 0.95 confidence intervals, * indicates significant factor differences, and # indicates significant differences between groups

**Fig. 5:**
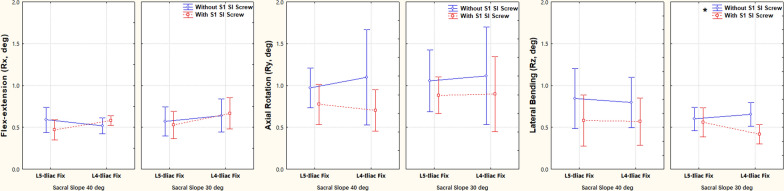
The effects of three factors, sacral slope (30° or 40°), additional iliosacral screw fixation (ULIF or TOS), and fixing level (L5 or L4–L5 fixation), on fracture rotation. *ULIF* Unilateral lumbar–iliac fixation, *TOS* triangular osteosynthesis. Vertical bars denote 0.95 confidence intervals, * indicates significant factor differences, and # indicates significant differences between groups

TOS significantly improved the pelvic stability compared with ULIF. Specifically, the TOS significantly reduced displacement in the left–right and vertical directions, total displacement, and lateral bending from 0.61 mm, 0.80 mm, 1.33 mm, and 0.84°, respectively, to 0.43 mm, 0.43 mm, 0.90 mm, and 0.42° (Figs. 4 and 5). At 40° SS, TOS significantly improved the stability of L5 ULIF and L4-5 ULIF in vertical displacement and the stability of L5 ULIF stability in total displacement (Fig. 4).

ULIF was more affected by the SS. At 30° SS, the displacement in the left–right and vertical directions, the total displacement, and the lateral bending were smaller than those at 40° SS, although the difference between SS was insignificant (Figs. 4 and 5). This also suggested that TOS had a greater role in limiting the fracture motion at larger SS.

Fixing level extended to L4 did not significantly improve stability at different SS, no matter in ULIF or TOS, and even led to more fracture motion (Fig. 4).

**Fig. 6:**
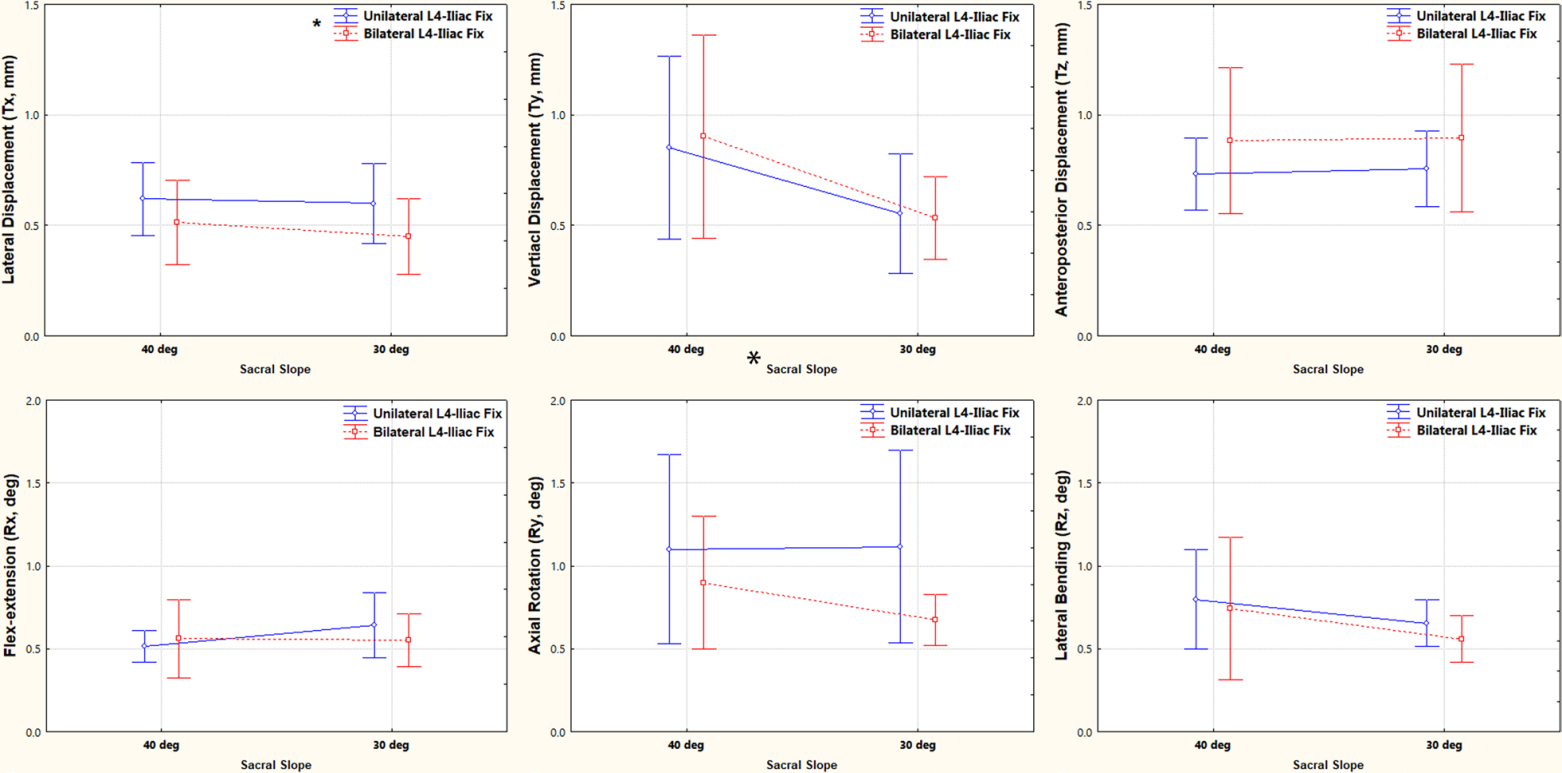
The effects of two factors, including fixing side (unilateral or bilateral) and sacral slope, on fracture displacement and rotation. Vertical bars denote 0.95 confidence intervals, * indicates significant factor differences, and # indicates significant differences between groups

When analyzing the effect of fixing side and the SS, we found that BLIF significantly limited left–right displacement when compared with ULIF (0.45 mm versus 0.60 mm). Vertical displacement of the L4–L5 ULIF was 0.85 mm at 40° sacral slope and significantly higher than that at 30° sacral slope (0.55 mm) (Fig. 6). The bilateral fixation also limited axial rotation and increased anteroposterior displacement without significant difference to the unilateral one. A comparison of L4–5 BLIF and L4–5 TOS was also performed, and the result showed that L4–5 TOS was solider than L4–5 BLIF vertically at 40° SS (*P* = 0.010).

## Discussion

The goal of treating Tile C1.3 pelvic fracture is to restore normal structure of the pelvic ring and rigidly fix it. For pelvic fractures that are difficult to reduce, especially the old fractures, LIF is an important surgical method for the treatment of Tile C1.3 pelvic fractures because of its strong reduction force and solid fixation. In this study, an in vitro biomechanical model of pelvic loading in two-legged stance was developed to factor analyze five LIF techniques, as well as sacral slope. The TOS fixation was shown to enhance the pelvic stability and significantly limited the motion in the coronal plane and cephalad–caudal direction, whereas L4–L5 ULIF did not further improve the immediate stability. BLIF significantly improved the stability in the left–right direction, but its vertical stability was significantly affected by the SS, suggesting that stronger fixation is required at greater SS.

The TOS fixation is a rigid triangular structure combined with the ULIF and the iliosacral screw, which provides multiplanar stability, facilitates early mobilization, reduces mortality, and protects the local neuroanatomical structures from additional injury [25–29]. However, these clinical advantages need to be confirmed by biomechanical studies, especially whether they are sufficiently stable in the basic functional posture of two-legged standing. The present study shows that TOS significantly enhanced the stability in all directions except motion in anterior–posterior direction or flexion–extension, supporting that TOS can provide solid instant stability for Tile C1.3 pelvic fracture.

Our study also shows that ULIF extended to L4 did not significantly improve the immediate stability, i.e., the stability of the L4–L5 ULIF and L4–L5 TOS was similar to that of the L5 ULIF and L5 TOS in the case of different SS. In the view of the principle of treatment of spinal fractures, both the upper and lower two segments of the fractured segment should be fixed to improve the reduction effect on the fracture and to minimize axial rotational motion. However, short fixation can surprisingly provide better stability and clinical efficacy than long fixation according to previous reports [30, 31]. The primary role of ULIF is to resist separation of the fractured posterior pelvic ring, which may explain the lack of stability-gaining effect of fixation extending to L4. In clinical practice, the L5 ULIF or the L5 TOS is enough to achieve strong fixation, which can reduce such complications as surgical invasion and postoperative infection.

This study further demonstrates that in the ULIF, the displacement in the left–right and vertical directions was significantly greater at 40° SS than that at 30° SS. However, the difference between two SS was not significant in either L4–L5 TOS or L5 TOS. We ascribed this phenomenon to the “central fixation” [32] of the iliosacral screw, which may improve the stability of the posterior pelvic ring in patients. L5 TOS is recommended for patients with a large SS to ensure the stability of fixation. In addition, studies have been performed to assess the effect of spinopelvic parameters on lumbar spine loading [33] and found that shear forces were greater at the L5–S1 segment at larger SS. We attribute the weakening of the vertical stability of the posterior pelvic ring with increasing sacral slope to the fact that increased lumbosacral shear requires stronger posterior ring fixation of the pelvic fracture, and additional iliosacral screw fixation can be effective in resisting lumbosacral shear. This study reveals for the first time the biomechanical influence of SS in posterior pelvic ring fixation, suggesting clinical attention to the fact that the vertical stability of posterior pelvic ring fixation is reduced in patients with higher sacral slope, which is also informative for postoperative rehabilitation.

The present study also demonstrated that the BLIF significantly limited left–right displacement when compared with the ULIF. From the perspective of spine biomechanics, bilateral fixation provides a more solid upper-end anchorage for Tile 1.3 pelvic fractures, which should increase the stability of the fracture fixation. However, the BLIF reduced the left–right displacement by only about 0.15 mm, and it is debatable whether it is of clinical significance. In terms of the magnitude of fracture displacement, the effect of SS appeared to be more pronounced, with the results of this study showing that a 10° increase in SS was associated with a 0.37 mm increase in vertical displacement. Both the L4–5 BLIF and the L4–5 TOS had a transversal connector between fracture fragments. The traversal connector of BLIF (the connecting rod) is eccentric fixation with small diameter and doubtful stability, while the one of TOS (the iliosacral screw) is central fixation with larger diameter and stronger stability. The biomechanical analysis also showed that BLIF could only increase the lateral stability compared with ULIF, while TOS could increase the total, vertical, and lateral one. The Tile C1.3 pelvic fracture lacks the total, vertical, and lateral stability, so the BLIF was not an ideal choice for this type of fracture, and we are cautious about it given its surgical costs and risks.

In a study by Roussouly et al. [34], 160 asymptomatic adult volunteers underwent full-length lateral X-rays of the spine in a standard standing position, and the mean sacral slope was 39.9 ± 8.2°. Moreover, in a similar study by Zhou et al. [35], the mean sacral slope was 34.0 ± 7.1°. Therefore, two angles of 30° and 40° were set in the present study to analyze the effect of sacral slope on lumbar–iliac fixation was appropriate, and it also confirmed the effect of pelvic parameters on the lumbar–iliac fixation.

The two-legged standing model used in this study is a validated biomechanical model which was originally developed by Hearn et al. [36] and has been used in many studies to assess the biomechanics of pelvic fixation effectiveness [10–13]. Specifically, the two-legged standing model is more suitable for biomechanical evaluation of pelvic fracture, as it indicates the physiological loading on the pelvis, where the load from the lumbar spine is transmitted through the sacrofemoral arch to the femur, and the pubic symphysis is distracted through the corresponding para-arch. In the present study, two metal femoral heads supported the bilateral acetabulum without affecting the pelvis motion during loading. The steel cables anchored to the anterior superior iliac spine anterior to the center of the femoral head adjust the SS, simulate the action of the hip flexors [8, 37], and maintain pelvic balance under loading.

In this study, fresh frozen cadaveric spinal–pelvic specimens with preserved major ligamentous provided a better representation of the in vivo condition [38] but with greater individual variability than artificial pelvic models with high homogeneity and better reproducibility [17]. A repeated-measures study design was used in this study, which reduced the effect of individual differences to some extent and still reflected the effects of different factors. Second, changing different internal fixations on the same specimen may affect the strength of the bone–screw interface. In this study, an experienced clinician placed the screws as close as possible to the original screw trajectory and achieved similar screw purchase during instrumentation. In addition, the 500 N axial compressive load was used and should not damage the bone–screw interface based on previous studies [39].

In conclusion, we developed an in vitro Tile C1.3 pelvic fractures model under two-legged standing load and confirmed that TOS enhanced the stability of LIF and significantly reduced the motion of the fracture fragments in the coronal plane and cephalad–caudal direction, whereas L4–L5 ULIF did not further improve the immediate stability. BLIF significantly improved the stability in the right–left direction, but its vertical stability was significantly affected by the SS. This study reveals the biomechanical impact of SS to LIF, suggesting that clinical attention needs to be paid to the vertical stability of posterior ring fixation of pelvic fractures in patients with higher sacral slope.

## Data Availability

Please contact author for data requests.

## Abbreviations

LIF: Lumbar–iliac fixation
ULIF: Unilateral lumbar–iliac fixation
BLIF: Bilateral lumbar–iliac fixation
TOS: Triangular osteosynthesis
SS: Sacral slope
